# Genome-wide investigation of calcium-dependent protein kinase gene family in pineapple: evolution and expression profiles during development and stress

**DOI:** 10.1186/s12864-020-6501-8

**Published:** 2020-01-23

**Authors:** Man Zhang, Yanhui Liu, Qing He, Mengnan Chai, Youmei Huang, Fangqian Chen, Xiaomei Wang, Yeqiang Liu, Hanyang Cai, Yuan Qin

**Affiliations:** 1 State Key Laboratory of Ecological Pest Control for Fujian and Taiwan Crops; Fujian Provincial Key Laboratory of Haixia Applied Plant Systems Biology; Key Laboratory of Genetics, Breeding and Multiple Utilization of Crops, Ministry of Education, Center for Genomics and Biotechnology, College of Plant Protection, College of life science, College of Agriculture, Fujian Agriculture and Forestry University, Fuzhou, 350002 Fujian Province China; 2 State Key Laboratory for Conservation and Utilization of Subtropical Agro-Bioresources, Guangxi Key Lab of Sugarcane Biology, College of Agriculture, Guangxi University, Nanning, 530004 China; 3 Horticulture Research Institute, Guangxi Academy of Agricultural Sciences, Nanning Investigation Station of South Subtropical Fruit Trees, Ministry of Agriculture, Nanning, 530007 China

**Keywords:** Pineapple, CPK, Genome-wide, Expression pattern, Stress

## Abstract

**Background:**

Calcium-dependent protein kinase (CPK) is one of the main Ca^2+^ combined protein kinase that play significant roles in plant growth, development and response to multiple stresses. Despite an important member of the stress responsive gene family, little is known about the evolutionary history and expression patterns of *CPK* genes in pineapple.

**Results:**

Herein, we identified and characterized 17 *AcoCPK* genes from pineapple genome, which were unevenly distributed across eight chromosomes. Based on the gene structure and phylogenetic tree analyses, *AcoCPKs* were divided into four groups with conserved domain. Synteny analysis identified 7 segmental duplication events of *AcoCPKs* and 5 syntenic blocks of *CPK* genes between pineapple and *Arabidopsis*, and 8 between pineapple and rice. Expression pattern of different tissues and development stages suggested that several genes are involved in the functional development of plants. Different expression levels under various abiotic stresses also indicated that the *CPK* family underwent functional divergence during long-term evolution. *AcoCPK1*, *AcoCPK3* and *AcoCPK6*, which were repressed by the abiotic stresses, were shown to be function in regulating pathogen resistance.

**Conclusions:**

17 *AcoCPK* genes from pineapple genome were identified. Our analyses provide an important foundation for understanding the potential roles of *AcoCPKs* in regulating pineapple response to biotic and abiotic stresses

## Background

In order to survive continual biotic and abiotic stresses occurred in the environment, plants have evolved an effective defense mechanism, including a variety of signal transduction pathways, especially the Calcium (Ca^2+^), which is a universal second messenger and can induce signal transduction in all eukaryotes. Particularly, plants can sense Ca^2+^ signaling to regulate growth, development, as well as responses to various biotic and abiotic stimuli [1, 2]. Plants possess several kinds of Ca^2+^ sensors, and many of them own the EF-hand motif, while the specific helix-loop-helix structure coordinates a single Ca^2+^ ion, providing direct Ca^2+^-binding ability to the sensors [3]. When plants were subjected to various stresses, Ca^2+^ sensors, such as calmodulin-like proteins (CaMLs), calmodulins (CaMs), and calcium-dependent protein kinases (CPKs), can sense and decoded the calcium fluxes concentration changes [4, 5]. In addition, the protein kinase and calmodulin-like domains of CPKs are located in a single polypeptide, resulting in Ca^2+^-binding and Ca^2+^-stimulated kinase activities within an independent protein product, which may arose direct translation of Ca^2+^ into downstream phosphorylation signals [6, 7].

Calcium-dependent protein kinases (CPKs) as a kind of ser/thr protein kinases, have been identified throughout the plant kingdom [6]. CPKs comprise four functional domains, including a serine/threonine kinase domain (STKD), an N-terminal variable domain (ND), an auto-inhibitory junction domain (AID) and a C-terminal regulatory calmodulin-like domain (CaM-LD) [6, 8]. The STKD is highly conserved, containing ATP binding catalytic domain and adjacent to the autoinhibitory junction domain [9]. The N-terminal domain consists myristoylation and palmitoylation sites, which are crucial for subcellular localization and molecular function and the two show the highest sequence divergence among CPK domains [10]. The AID, which may sometimes be part of the CaM-LD [9], contains a pseudo-substrate sequence so as to interact with the active site or inhibit kinase activity. The calmodulin-like domain contains one to four EF-hand structures for Ca^2+^ binding [8]. Because of these unique features, the CPKs is sensitive to Ca^2+^ and play an important role in regulating the downstream components of calcium signaling pathway.

Recently, genetic evidences indicates that *CPK* genes are ubiquitously functional in plant growth and developmental process such as flowering [11], pollen tube growth [11], fruit development [12], root development [13], cell division and differentiation, and cell death [14]. CPKs are also involved in abiotic and biotic stress responses. In *Arabidopsis*, AtCPK4/11/21, as positive regulators in the ABA signaling processes, were involved in resistance to drought and salt stresses [15, 16]. *CPK* gene from maize, such as *ZmCPK4*, also has similar functions in the responses to salt and drought stresses [17]. Furthermore, *OsCPK12*, which is involved in the ABA signaling process, improved salt resistance through a reduction in ROS accumulation [18]. The expression of *OsCPK13* can be induced under low temperature [19], however, *ZmCPK1* plays as a negative regulator in response to cold stress [20]. Overexpressing the *OsCPK7* gene enhanced tolerance of transgenic plants to drought, salt, and cold stresses [21]. The recombinant protein StCPK7, an active Ca^2+^-dependent protein kinase, functions in plant defense response and can be induced upon infection with *Phytophthora infestans* in potato [22]. *VaCPK20* gene overexpression significantly increased resveratrol content of *Vitis amurensis Rupr* [23].

The genes encoding CPKs form a multi-gene family and they have been well characterized in many plant species. To date, genome-wide analyses have identified 34 *CPK* genes in *Arabidopsis* [8], 31 *CPK* genes in rice [24], 30 *CPK* genes in poplar [25], 20 *CPK* genes in wheat [26], 41 *CPK* genes in cotton [27], 29 *CPK* genes in tomato [28], and 19 *CPK* genes in cucumber [29]. Nevertheless, our knowledge of *CPK* gene family for many other economically important horticultural crops, such as pineapple (*Ananas comosus*), is still limited. Like other economical plants, pineapple is often affected by various abiotic and biotic stresses such as salt, drought, pathogens and so on. The decoding of the pineapple genome sequencing provided a chance to reveal the organization, expression and evolutionary characterization of pineapple CPK genes at the genome-wide level [30]. In this study, a total of 17 *CPK* genes were found and these *CPKs* were grouped based on their phylogenetic relationships into four subgroups and were located to specific chromosomes. Our study concluded the exon-intron organization, motif compositions, gene duplications, phylogenetic and synteny relationships of pineapple *CPKs*. Global expression analyses were also performed to identify involvement of specific pineapple *CPK* genes in different tissues and various stresses. This work provides insights into the evolutionary history and biological functions of pineapple *CPK* family.

## Results

### Identification of *CPK* genes in pineapple genome

A total of 17 putative CPK genes were identified from the pineapple genome, and named from *AcoCPK1* to *AcoCPK17* (Additional file 4: Table S1). The full-length of 17 CPK proteins varied from 303 (AcoCPK17) to 578 (AcoCPK13) amino acid residues with CDS ranging from 912 to 1737 bp, and relative molecular mass distributing from 34.45 to 65.23 kDa, following with isoelectric points ranged from 4.91 to 8.25. All of them contain the typical CPK structure, including an N-variable domain, a protein kinase domain, an autoinhibitory domain, and a CaM-like domain. In addition, all the pineapple *CPK* genes exist four EF-hand motifs in the CaM-like domain by predicting, which can recognize and bind Ca^2+^ molecules (Additional file 4: Table S1, [8, 31]. Among the identified 17 pineapple CPK proteins, 6 CPKs were predicted to contain myristoylation motifs at their N-termini (Additional file 4: Table S1).

### Phylogenetic analysis, gene structure of *CPK* genes and their chromosomal location

**Fig. 1:**
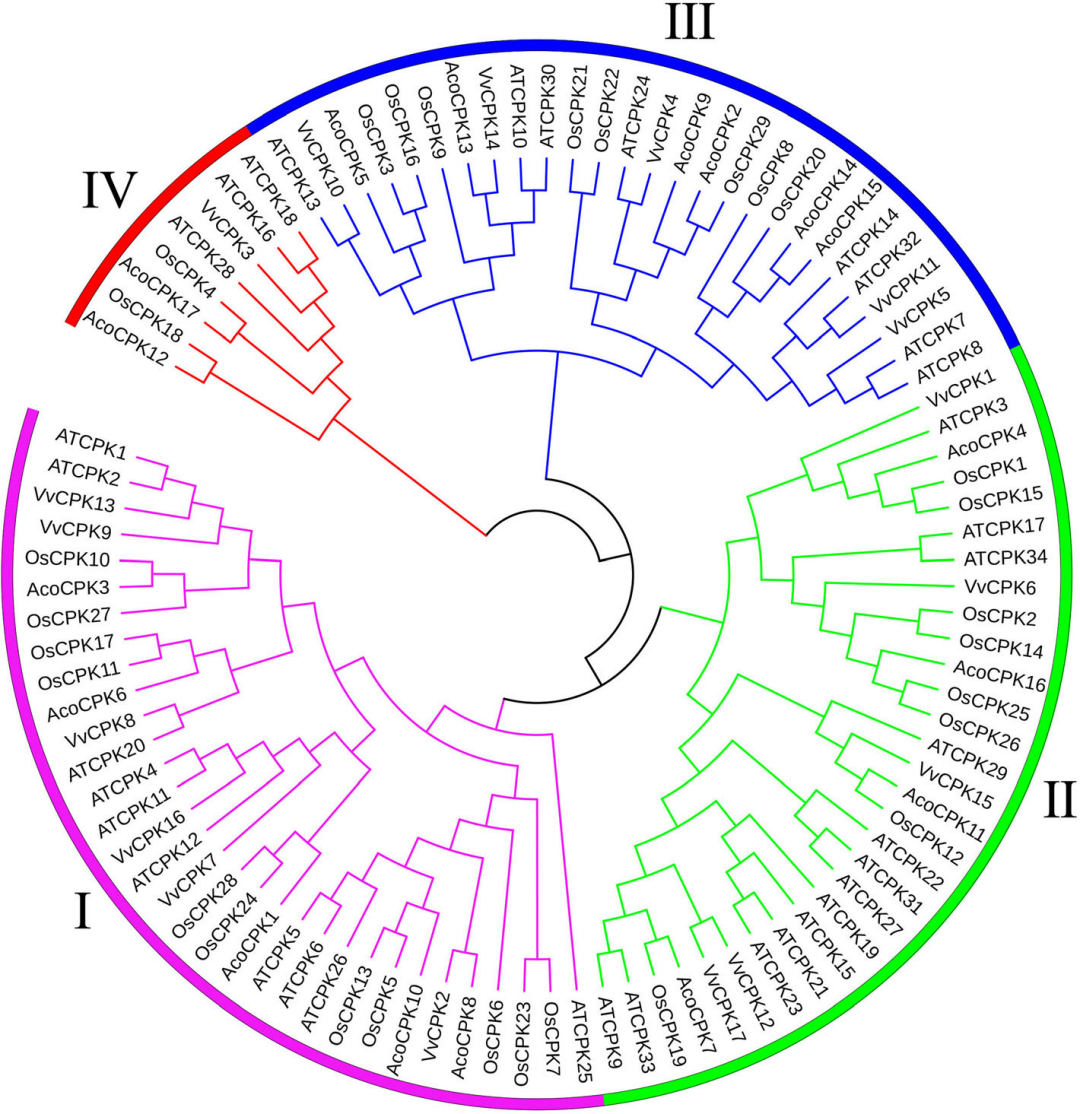
Unrooted phylogenetic tree representing relationships among CPK domains of pineapple, *Arabidopsis* and grape. The different-colored arcs indicate different groups of CPK domains

To examine the phylogenetic relationship among the CPKs in pineapple, the CPKs of four species, including pineapple, *Arabidopsis*, grape and rice, were constructed using MEGA5.0. *CPK* genes were grouped into four subfamilies, including 5, 4, 6 and 2 members in group I, II, III, and IV, respectively (Fig. 1 and Additional file 4: Table S1).

**Fig. 2:**
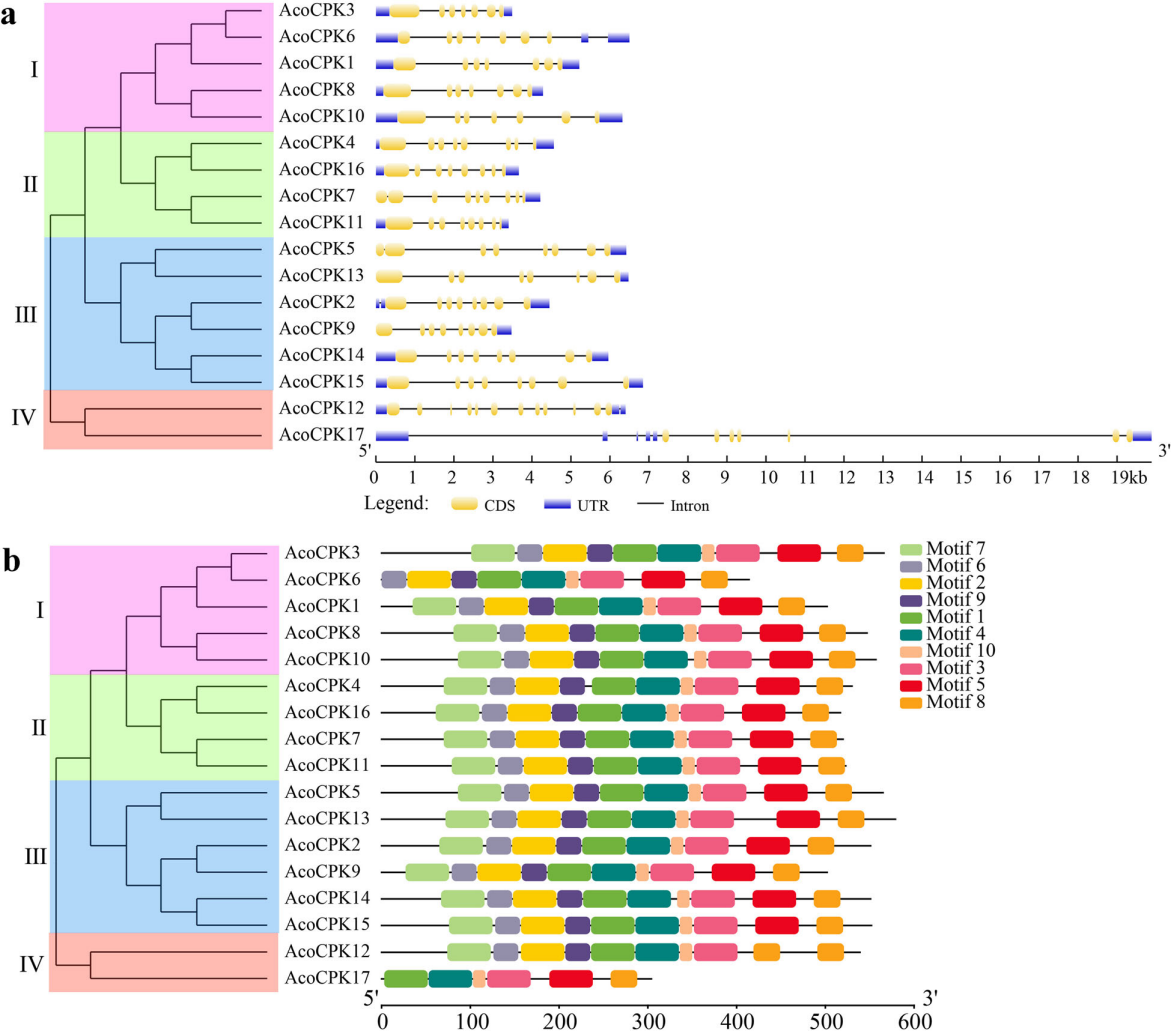
Phylogenetic relationships, gene structure and architecture of conserved protein motifs in *CPK* genes from pineapple. The phylogenetic tree was constructed based on the full-length sequences of pineapple CPK proteins using MEGA 5 software. Details of clusters are shown in different colors. a Exon-intron structure of pineapple *CPK* genes. b Motif composition of pineapple CPK proteins. The motifs, numbers 1–10, are displayed in different colored boxes. The length of protein can be estimated using the scale at the bottom

To obtain the possible structural evolution of *CPK* genes in the pineapple genome, diverse exon-intron organizations of *AcoCPKs* were compared. As shown in Fig. 2a, all *AcoCPK* genes possesses six to eleven introns (four with six introns, 10 with seven introns, one with eight introns, and two with eleven introns). Genes in the same subfamily shared very similar exon-intron structures. All members of group I possessed seven exons. In subfamily II, diverse numbers of exons were found in different members: eight exons were found in *AcoCPK4*, *AcoCPK16* and *AcoCPK11*, nine exons were found in *AcoCPK7*. Compared with the Group I members, Group II members have one or two additional exons. In group III, all members had eight exons. The two members in group IV had 12 and 7 exons with 11 introns. The results indicate that *CPK* genes with higher homogenous sequences tend to have the same numbers of exons. A schematic representing the structure of all AcoCPK proteins was constructed from the MEME motif analysis results. As exhibited in Fig. 2b, a total of 10 distinct conserved motifs were found (Additional file 1: Figure S1), almost all the CPK family members harbor ten motifs, except for AcoCPK6 in group I without motif 7, AcoCPK12 and 17 in group IV without motif 5 and motif 7/2/6/9. In conclusion, group IV may be the most conserved and presented earliest.

**Fig. 3:**
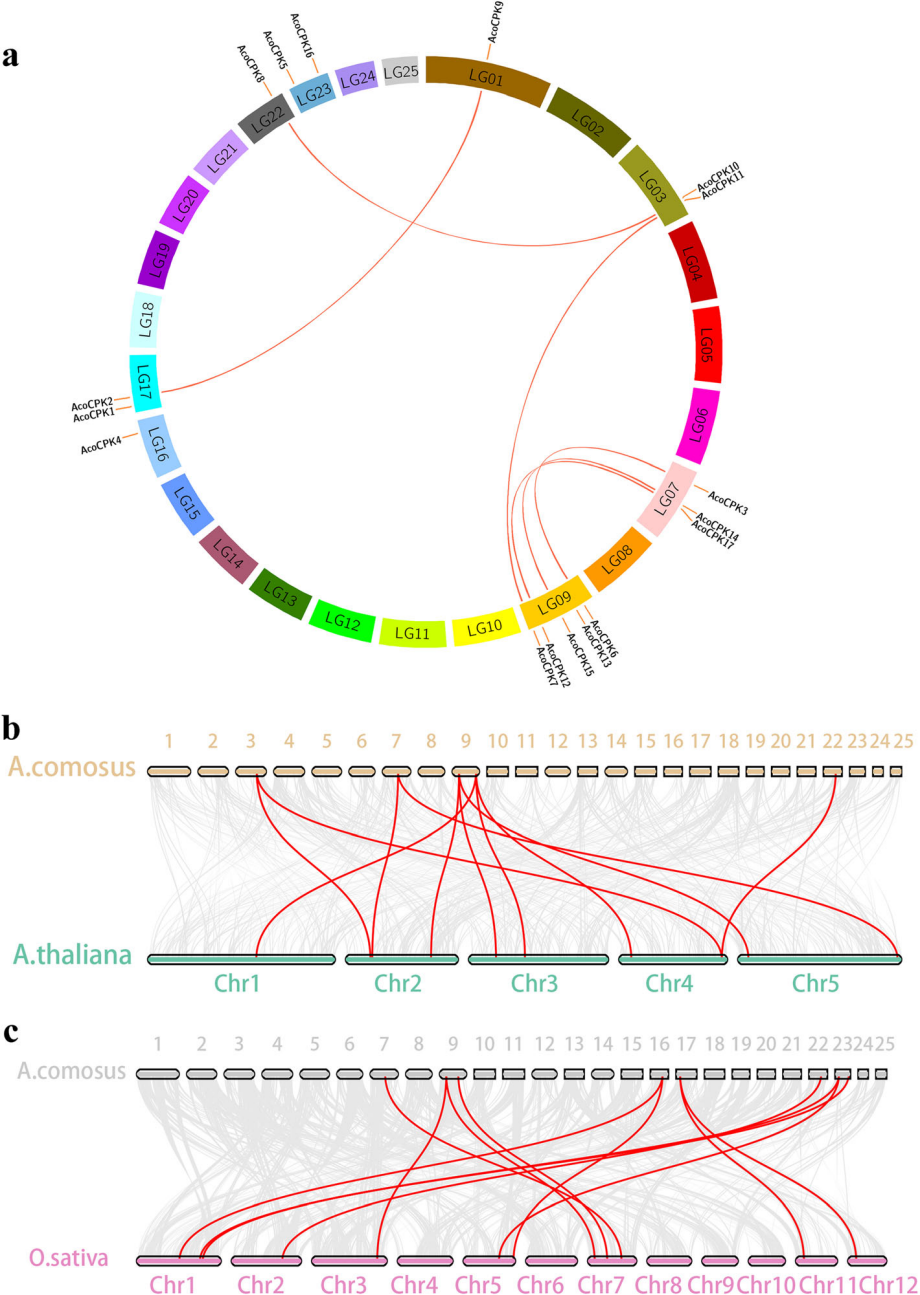
Synteny analysis of *CPK* genes between pineapple and two representative plant species. **a** Schematic representation for the chromosomal distribution and interchromosomal relationships of pineapple *CPK* genes. **b** Synteny analysis of *CPK* genes between pineapple and *Arabidopsis*. **c** Synteny analysis of *CPK* genes between pineapple and rice. Gray lines in the background indicate the collinear blocks within pineapple and other plant genomes, while the red lines highlight the syntenic *CPK* gene pairs

All of 17 pineapple *AcoCPK* genes were mapped onto eight chromosomes (Fig. 3). Some chromosomes have more genes, whereas others have few: the largest numbers of *CPK* genes (five) were located to chromosome 9; 3 *CPK* genes were located to chromosome 7, and chromosomes 3, 17 and 23 were found to harbor two *CPK* genes each. Chromosome 1, 16 and 22 were each found to harbor one *CPK* gene.

### Synteny analysis of pineapple *CPK* genes

To elucidate the expanded mechanism of the *CPK* gene family in pineapple, gene duplication events, including tandem and segmental duplications, were investigated. A total of 7 duplicated *CPK* gene pairs, *AcoCPK3*/*AcoCPK6*, *AcoCPK8*/*AcoCPK10*, *AcoCPK7*/*AcoCPK11*, *AcoCPK2*/*AcoCPK9*, *AcoCPK14*/*AcoCPK15*, *AcoCPK5*/*AcoCPK13*, and *AcoCPK12*/*AcoCPK17*, were found in the pineapple genome; all of these were segmental duplicates (Fig. 3a, Additional file 5: Table S2). The result suggested that segmental duplication played an important role in the amplification of *CPK* gene family members in the pineapple genome.

In order to infer the evolutionary mechanism of pineapple CPK family, we constructed two comparative syntenic maps of pineapple associated with *Arabidopsis* and rice (Fig. 3b, c). A total of five *AcoCPK* genes showed syntenic relationship with those in *Arabidopsis*, eight in rice (Additional file 6: Table S3, Additional file 7: Table S4). Between *Arabidopsis* and pineapple *CPK* genes, we could find several kinds of syntenic orthologous gene pairs: one pineapple gene vs multiple *Arabidopsis* genes, such as *AcoCPK6-ATCPK1/2/20*, *AcoCPK7-ATCPK9/21/33*; one *Arabidopsis* gene vs multiple pineapple genes, such as: *ATCPK26*-*AcoCPK8/10* (Fig. 3b, Additional file 6: Table S3). Between rice and pineapple *CPK* genes, four pairs of syntenic orthologous genes (one to one) were identified: *AcoCPK8-OsCPK5*, *AcoCPK12-OsCPK18*, *AcoCPK14-OsCPK20* and *AcoCPK10-OsCPK2* (Fig. 3c, Additional file 7: Table S4), indicating that these genes might be derived from the same ancestor of rice and pineapple. We also found that one pineapple gene corresponds to multiple rice genes, such as *AcoCPK1-OsCPK24/28*. Interestingly, some orthologous gene pairs mapped between pineapple and rice were not found between pineapple and *Arabidopsis*, such as *AcoCPK5*-*OsCPK3/16*, which may indicate that these orthologous pairs formed after the divergence of dicotyledonous and monocotyledonous plants. For further evolutionary studies, the Ka, Ks and Ka/Ks of the orthologous gene pairs were calculated based on the comparative synteny map (Additional file 6: Table S3, Additional file 7: Table S4). The majority of orthologous *CPK* gene pairs had Ka/Ks < 1, suggesting that the pineapple *CPK* gene family might have experienced strong purifying selective pressure during evolution.

### Pineapple CPK genes are expressed in different tissues in pineapple plants

To investigate the possible roles of the *CPK* genes in the pineapple genome, we analysis the expression profiles of the 17 *CPK* genes in different tissues and developmental stages using RNA-seq expression data recently published by Ming et al. from MD2 pineapple plants (Additional file 2: Figure S2 and Additional file 8: Table S5, [30]. The results showed that all the *CPK* genes were expressed in different tissues and developmental stages in pineapple. Some genes showed preferential expression across the detected tissues. Remarkably, *AcoCPK16* showed high expression level in flower and leaf while barely any expression in root and different stage fruits, and *AcoCPK2*, *AcoCPK6* and *AcoCPK9* also had high expression level in flower and leaf but lower than *AcoCPK16*, and *AcoCPK2*, *AcoCPK6* and *AcoCPK9* showed similar expression pattern. On the contrary, *AcoCPK7* displayed high expression level in different development stage of fruit, indicating they might participate in the maturity process of pineapple fruit. Besides, *AcoCPK12*, *AcoCPK4*, *AcoCPK10* and *AcoCPK14* showed similar expression level in different tissues and fruits in different stages, suggesting they might be constitutive expression in pineapple and involved in different development stages. All these data suggest that the members of the *CPK* gene family might be involved in the growth and development of different tissues or organs of pineapple.

### Expression profiles of pineapple *CPK* genes in response to different treatments

To explore the mechanisms of *CPK* response to the abiotic stresses, we searched for 15 stress-related *cis-elements* in the *AcoCPK* promoters, such as W-box, HSE and MBS, (Additional file 9: Table S6). The results showed that more than one different *cis-elements* located in the promoters of all 17 *CPK* genes with the least 4 *cis-elements* in the promoters of *AcoCPK1* and more than 9 *cis-elements* in the promoter of *AcoCPK12* and *AcoCPK15*. Some elements were detected more than one copy in the promoter regions. For example, the promoter of *AcoCPK3* contained 4 copies of MBS sequences and the promoter of *AcoCPK12* contained 4 copies of W-box sequences. At least one MBS was present in 94% (16 out of 17) of *AcoCPK* promoters, indicating that MBS plays a crucial role in response to stress in pineapple.

**Fig. 4:**
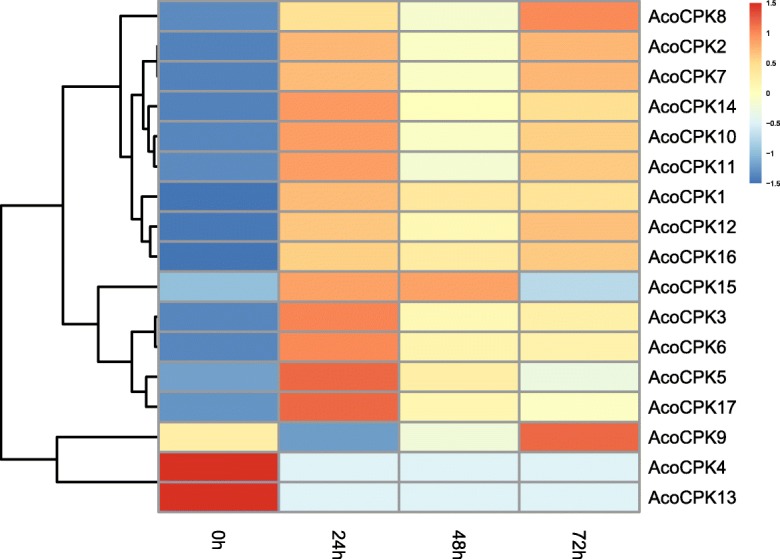
Expression profile of the pineapple *CPK* genes under mealybugs infection

**Fig. 5:**
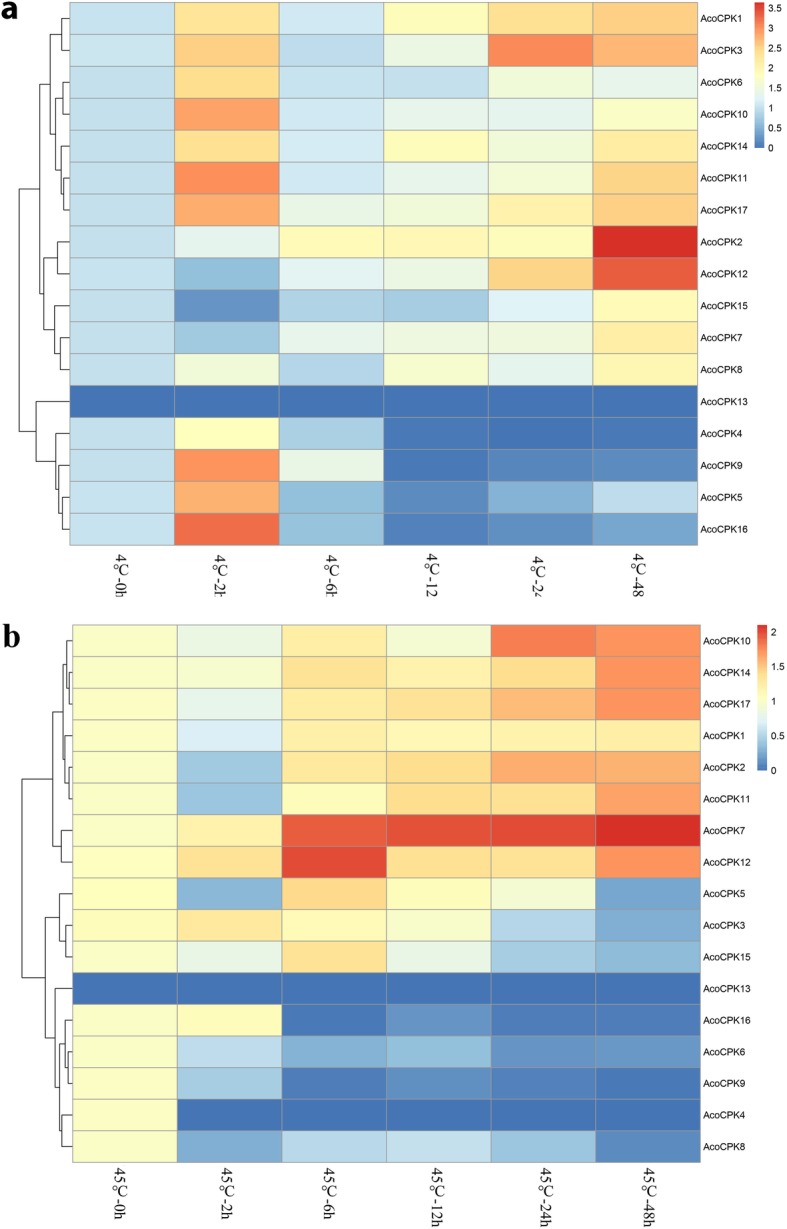
Expression analysis of pineapple *CPK* genes under cold **a** and heat **b** stress treatments

**Fig. 6:**
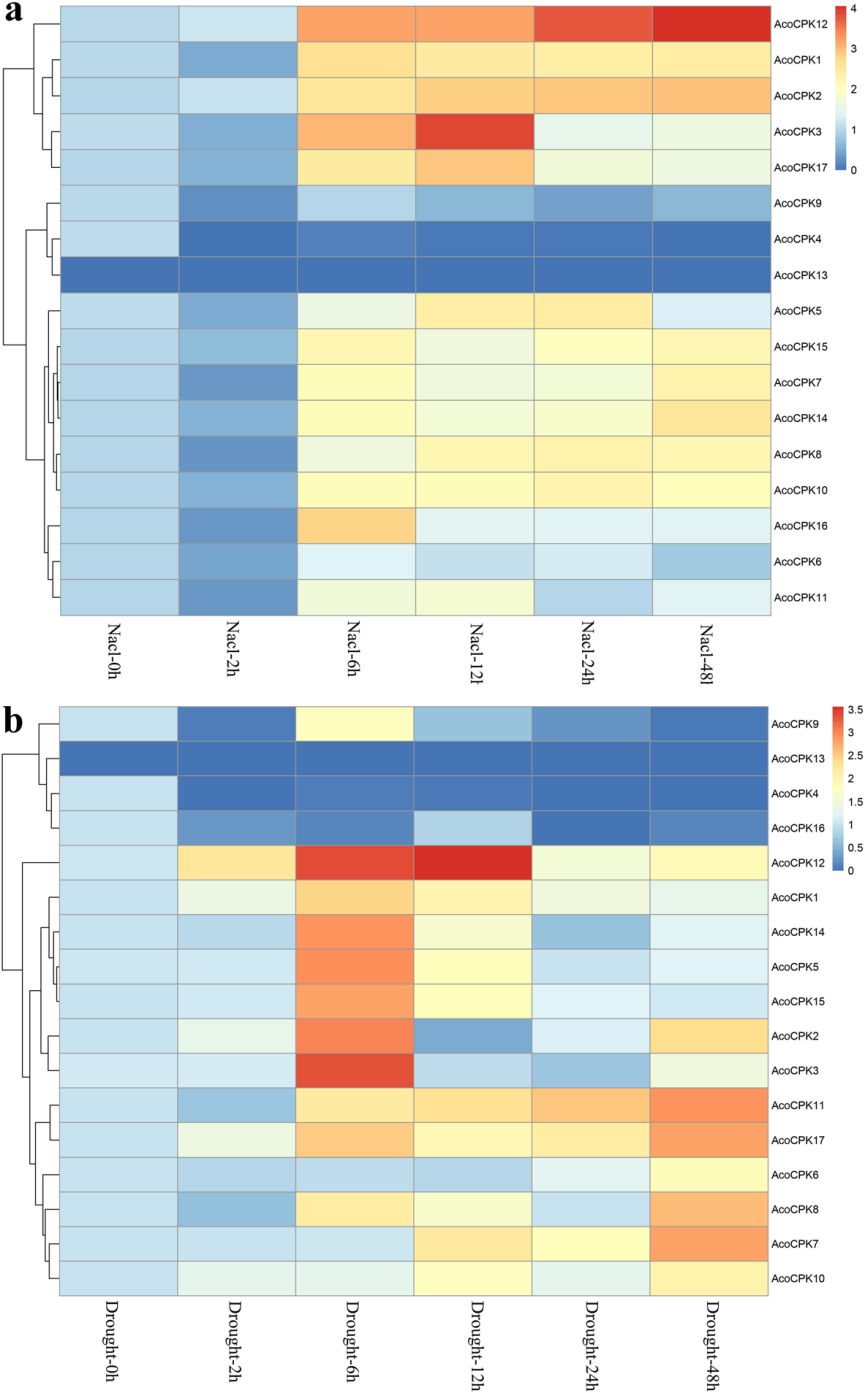
Expression analysis of pineapple *CPK* genes under salt **a** and drought **b** stress treatments

To further confirm whether the expression of *AcoCPK* genes were influenced by different abiotic stresses, qRT-PCR experiments were performed to analysis the *CPK* gene family members expression patterns in response to different treatments, including cold, heat, salt stress, drought stress and (*Dysmicoccus brevipes*) infection (Figs. 4, 5 and 6, Additional file 10: Table S7, Additional file 11: Table S8, Additional file 12: Table S9, Additional file 13: Table S10 and Additional file 14: Table S11). When response to biotic stress, most members were induced by mealybugs, except for *AcoCPK4* and *AcoCPK13*. Some *AcoCPK* genes were induced at early stage of infection (24 h) and then downregulated continuously, such as *AcoCPK1/3/6*; some genes showed highest expression level at 72 h, such as *AcoCPK7/9*. Overall, we found all family members had responses to all the abiotic treatments, except for *AcoCPK13*. some *AcoCPK* genes were induced/repressed by multiple treatments. For instance, *AcoCPK2/12* were significantly induced by all tested treatments, while *AcoCPK4* was repressed by all tested treatments. Upon these stresses, some genes were suppressed at 2 h and then upregulated continuously, such as *AcoCPK1/14/15* response to salt stress, *AcoCPK1/11* response to heat stress, so they might be crucial for later stage of stress responses; some genes were upregulated until 6 h after that they were suppressed, such as *AcoCPK5/14* response to drought stress, so they might play an important role at early stage of stress responses. Interestingly, some *AcoCPK* genes showed opposing expression patterns under different treatments. For instance, *AcoCPK11* was suppressed at 2 h and then upregulated continuously when response to salt, drought and heat stress, but it showed opposite expression pattern facing cold stress.

### Function analysis of *AcoCPK1*, *AcoCPK3* and *AcoCPK6*

**Fig. 7:**
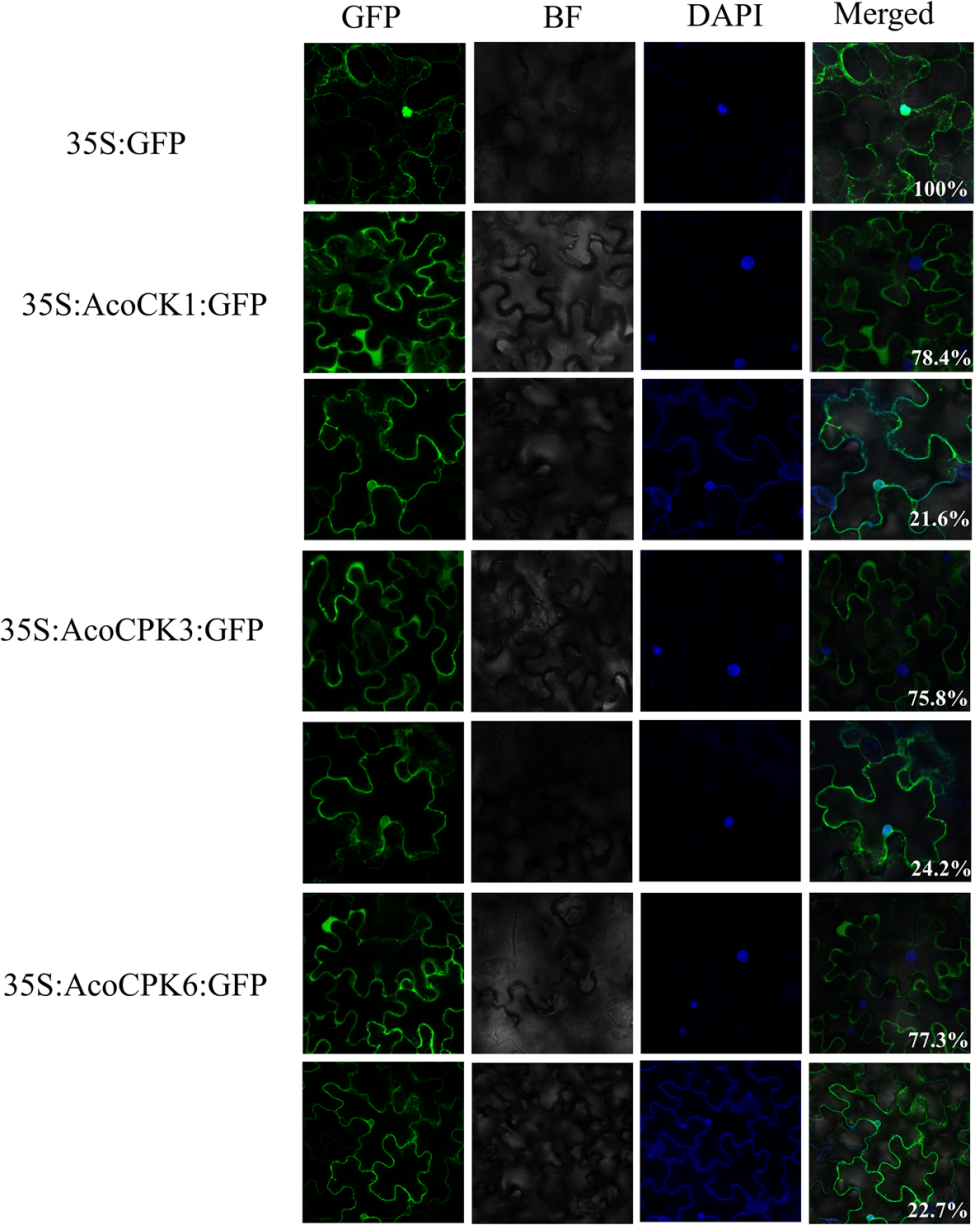
subcellular location of AcoCPK1, AcoCPK3 and AcoCPK6

**Fig. 8:**
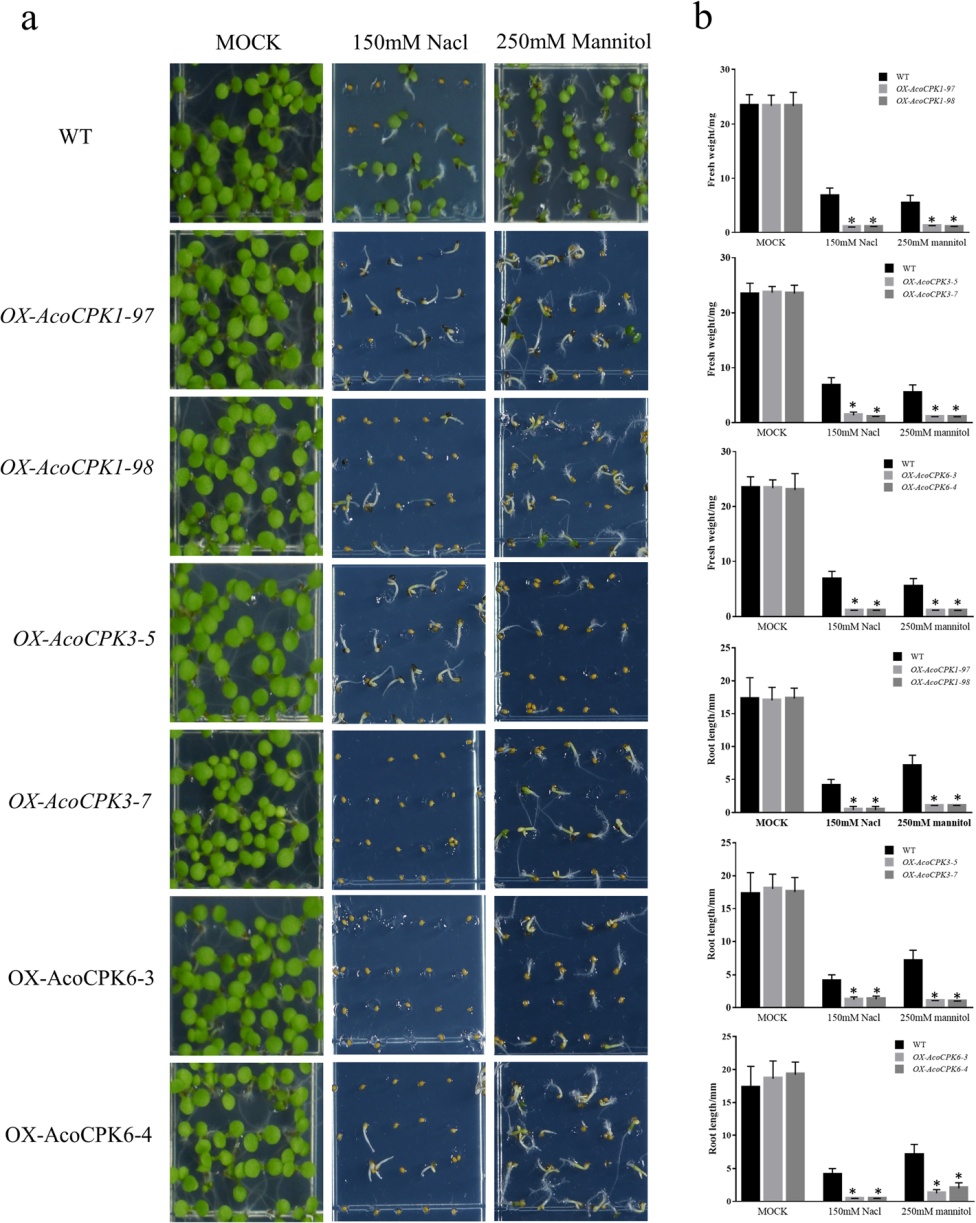
Effect of salt and drought stress on transgenic *Arabidopsis* plants overexpression *AcoCPK1*, *AcoCPK3* and *AcoCPK6*. **a** Representative image of 5-day-old seedlings of wild-type and *AcoCPK1*, *AcoCPK3* and *AcoCPK6* overexpression transgenic lines under salt and drought stress treatments. **b** Root length and fresh weight of 5-day-old seedlings of wild-type and *AcoCPK1*, *AcoCPK3* and *AcoCPK6* overexpression transgenic lines under salt and drought stress treatments

The expression pattern of pineapple *CPK* family showed that the members of *CPK* play crucial role in response to different abiotic and biotic stress. In order to further investigate their function, three group**I**members (*AcoCPK1/3/6*) were selected for the further research. To investigate the subcellular location of *AcoCPK1*, *AcoCPK3* and *AcoCPK6*, the coding regions of these genes were fused with GFP and transiently expressed in *Nicotiana benthamiana* leaves. The control vector (35S::GFP)-transformed leaves displayed GFP in both cell nuclei and membrane. Interestingly, we found that about 80% GFP signals of AcoCPK1-GFP, AcoCPK3-GFP and AcoCPK6-GFP were predominantly localized at cellular membranes, while some signals (~ 20%) were co-localized with DAPI-stained cell nuclei in the infiltrated leaf areas (Fig. 7). To determine the role of these three genes in response to abiotic and biotic stresses, we generated *AcoCPK1*, *AcoDCPK3* and *AcoCPK6* overexpression transgenic *Arabidopsis* plants. For each gene, two independent homozygous lines with relative high expression of transgenes were selected for further research (Additional file 3: Figure S3). Under normal condition, all transgenic lines showed no significant phenotypic differences with the wild type (Columbia-0). Under salt and drought stress conditions, overexpression lines of *AcoCPK1*, *AcoCPK3* and *AcoCPK6* are more sensitive to the salt and D-mannitol. As shown in the Fig.8, *Arabidopsis* plants that overexpress *AcoCPK1*, *AcoCPK3* and *AcoCPK6* showed much reduced seed germination ratios or green cotyledon under stress conditions (Fig.8a), and their root length and fresh weight were lower than wild type (Fig.8b).

**Fig. 9:**
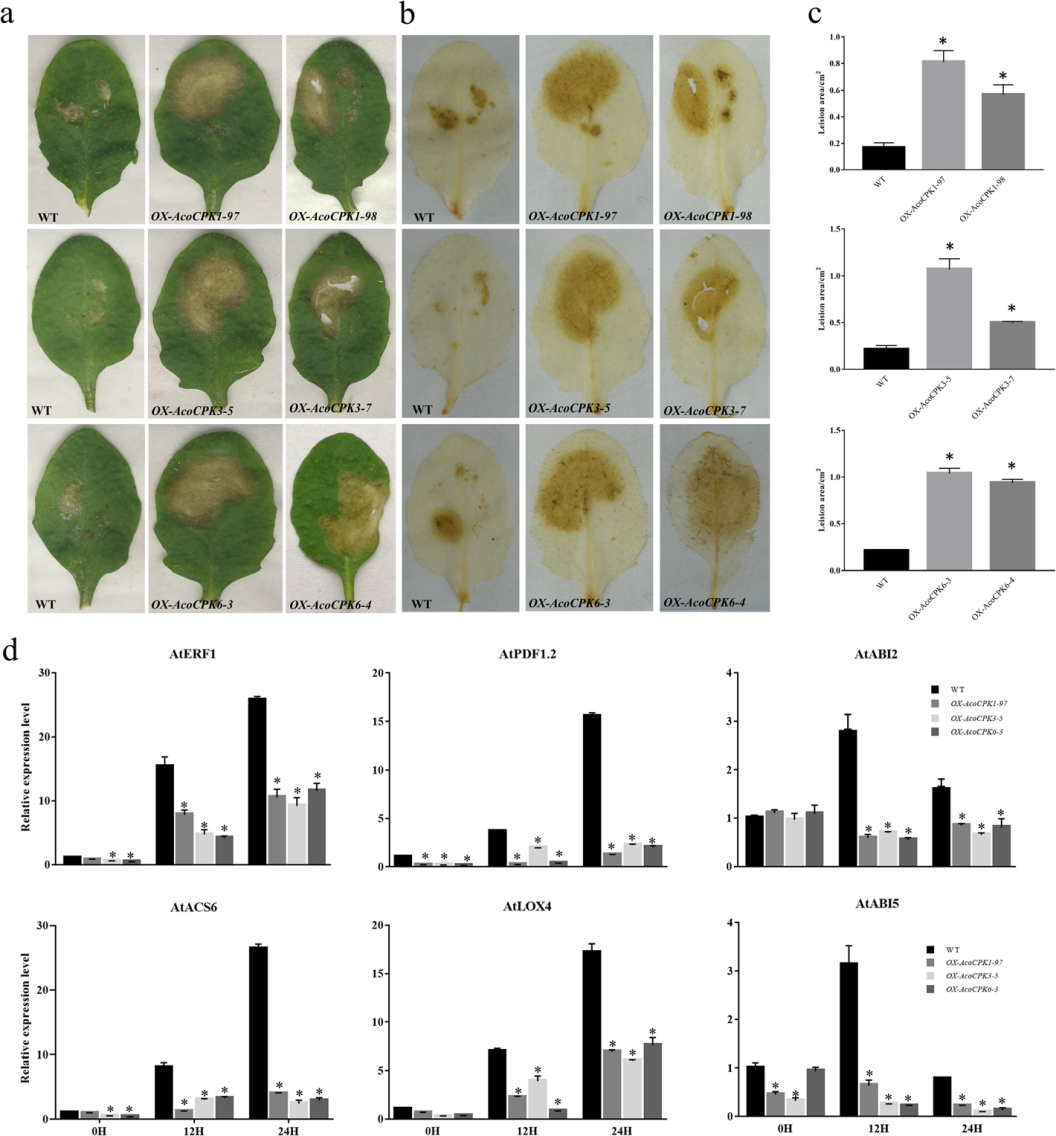
Overexpression of *AcoCPK1*, *AcoCPK3* and *AcoCPK6* confer susceptible disease resistance in *Arabidopsis* against *S. sclerotiorum*. **a** Three weeks old *Arabidopsis* leaves of wild-type and *AcoCPK1*, *AcoCPK3* and *AcoCPK6* overexpression transgenic lines were challenged with *S. sclerotiorum*, 24 h post-inoculation. **b** DAB staining of the *Arabidopsis* leaves after 24 h inoculation of *S. sclerotiorum*. **c** Lesion area resulting from *S. sclerotiorum* after 24 h inoculation. **d** Expression patterns of biotic stress related marker genes of wild-type and *AcoCPK1*, *AcoCPK3* and *AcoCPK6* overexpression transgenic lines

Previous studies have shown that the expression of many *CPK* genes be induced by biotic and abiotic stresses in *Arabidopsis* and rice [18, 32], so we checked the function of these three *CPK* genes upon plant disease resistance. The leaf surface of WT, OX-*AcoCPK1*, *OX-AcoCPK3* and *OX-AcoCPK6* were infected with *S. sclerotiorum*. After 24 h inoculation of *S. sclerotiorum*, *AcoCPK* gene overexpression lines are more sensitive to *S. sclerotiorum*, and the lesion areas are bigger than wild type, and they can generate more H_2_O_2_ (Fig.9a, b and c). As we know, phytohormones play key roles in local and systemic acquired resistance (SAR) to necrotrophic pathogens, such as jasmonic acid (JA), ethylene (ET) and abscisic acid (ABA). Disease resistance marker genes, *PDF1.2* and *LOX4*, which are related to JA, have been suggested to be involved in the plant’s defense pathway [33, 34]; *ACS6* and *ERF*, which is related to ET, are function in several necrotrophic fungi resistance [35, 36]; *ABI2* and *ABI5*, which is related to ABA, can response to the plant disease [37]. We checked expression pattern of these marker genes that response to these phytohormones and found that all of them were downregulated significantly in the *AcoCPK* gene overexpression lines compared with wild type (Fig.9d), coinciding with the reduced resistance to pathogen in the *AcoCPK1, AcoCPK3* or *AcoCPK6* overexpression lines.

## Discussion

Pineapple (*Ananas comosus*) is a tropical plant and the most economically significant plant in the *Bromeliaceae* family. *CPK* genes play important roles in diverse plant developmental and physiological process, as well as various plant biotic and abiotic stress responses. In the current study, a search for *CPK* genes in the pineapple genome resulted in identification of 17 members, which named from *AcoCPK1* to *AcoCPK17* on the basis of their chromosomal location, together with an analysis of their structure, evolutionary history and expression diversity with respect to biotic and abiotic stresses.

Evolutionary analysis indicated that *CPK* genes in pineapple can be divided into four groups, and same evolutionary classification was also found in other species, such as *Arabidopsis*, grape and rice [8, 10, 24]. In addition, the classification result was further confirmed by gene structure and conserved motif analyses. In pineapple, the number of introns changed from 6 to 11, which is similar with melon and pepper [38, 39], indicating that different species display similar gene structure diversity of *CPK* genes. According to a previous report, the rate of intron loss is faster than the rate of intron gain after segmental duplication in rice [40]. We also observed that groupIVin pineapple own more number of introns, indicating that group IV might contain the original genes, and this conclusion can be further supported by the evidence that motifs in group IV were the most conserved.

Most of the CPK proteins were slightly acidic in terms of biochemical properties, with isoelectric points (pI) ranging 5–7 [38, 39]. However, a few CPK proteins mainly distributed group IV, had basic pIs of 8 or more [6, 41]. In our research, CPK proteins in group Iwere slightly acidic with PIs ranging from 4.91 to 5.83. However, the other three groups all had member with PIs more than 7, such as AcoCPK7 in groupII, AcoCPK14/15 in group III and AcoCPK12 in groupIV (Additional file 5: Table S2). In the previous research, people have found that CPK proteins which own less than four EF-hands, have been found broadly in both monocotyledon and dicotyledon, such as *Arabidopsis* soybean, maize and rice [8, 42–44]. According to gene structure investigation, we found that all 17 pineapple *CPK* genes contain four EF-hands (Additional file 4: Table S1), which is similar to that of barley and pepper [39, 41].

In our study, phylogenetic relationships were investigated to illuminate the evolutionary history of the *CPK* gene family. Our results demonstrated the origin of the *CPK* genes before the divergence of eudicot and monocot with the result that all *CPK* genes from pineapple, *Arabidopsis*, grape and rice were distributed among all four groups [38, 39, 45]. We compared the number of *CPK* genes in pineapple with other sequenced eudicot and monocot genomes, including *Arabidopsis*, soybean, rice and grape, and found that pineapple possesses less number of genes [8, 24, 39, 42, 46]. Compared the genome with the basal angiosperm *Amborella*, people found that the grasses underwent three whole-genome duplication (WGD) events (τ, σ, and ρ) [30, 47], while pineapple only underwent two genome doublings (τ and σ) [30]. This might the reason that pineapple possesses the smaller number of CPK genes. Gene duplication events play a vital role in genomic rearrangements and expansions [48] and are defined as either tandem duplications or segmental duplications depending on duplicated genes on the same or different chromosomes [49]. In our research, chromosomal locations and duplication events implied that gene duplication, especially segmental duplication was the major evolutionary mechanism resulting in pineapple CPK expansions (Fig.3a). We found that the two genes in the same gene pair tended to be clustered into one group, such that segmental pairs *AcoCPK3*-*AcoCPK6*, *AcoCPK2*-*AcoCPK9* and *AcoCPK12*- *AcoCPK17* were clustered to group I, III and IV, respectively.

The synteny analysis illuminates the relevance between functional and evolutionary in two species, such as pineapple and *Arabidopsis* or rice syntenic genes. Many *Arabidopsis* and rice *CPK* genes response to biotic and abiotic stresses have been reported. For example, *CPK5*/*CPK6* in *Arabidopsis* are involved in the wounding-induced ethylene biosynthesis via differential regulation of *ACS* genes at transcriptional level [50]. As a component of the innate immune system of *Arabidopsis* plants, loss-of-function mutants of *AtCPK1* exhibit higher susceptibility to pathogen infection compared to wild-type plants [32]. In *Arabidopsis*, *AtCPK3* plays extensive roles in various biological and environmental responses [51]. *OsCPK12*-overexpressing plants exhibited increased tolerance to salt stress and increased susceptibility to blast fungus [18]. Overexpression of *OsCPK7* enhanced induction of some stress-responsive genes in response to salt/drought, but not to cold [21]. A total of 5 pineapple genes and 11 *Arabidopsis* genes were identified as syntenic orthologs between pineapple and *Arabidopsis*, 8 pineapple genes and 12 rice genes were identified as syntenic orthologs between pineapple and rice and the diversity of gene combinations reveals complex regulatory relationships. Some *CPK* genes present in these species, including pineapple, *Arabidopsis* and rice, could not be mapped into any syntenic gene pairs, it might due to multiple rounds of important chromosomal rearrangement and fusions happened in their genomes since the speciation of pineapple and *Arabidopsis* or rice, followed by selective gene loss, which can obscure the identification of chromosomal syntenies [49, 52].

In non-stressed pineapple plants, about half of *CPK* family members were expressed wildly in almost all the tissues and developmental stages according to pineapple expression pattern (Additional file 2: Figure S2). The duplicate genes may have different transcription levels in different tissues, development stages and even response to various stresses. The gene pair *AcoCPK3* and *AcoCPK6*, for example, had different transcriptional levels in different tissues: *AcoCPK6* displayed high expression level in leaf and flower, while *AcoCPK3* showed opposite expression profile. Specifically, *AcoCPK6* was down-regulated, whereas *AcoCPK3* was up-regulated and reached peak at 12 h dpi under hot stress, but these two genes were sensitive to the salt and drought stress (Figs.5, 6 and 8). These results indicated that the plants CPKs have evolved more specified functions through gene divergence to meet a broader array of lineage-specific requirements.

To understand the specific functions of pineapple CPKs, we randomly selected eight CPK genes for function analysis and the genes *AcoCPK2*, *AcoCPK4*, *AcoCPK7*, *AcoCPK9 and AcoCPK12* could not be cloned by PCR. We found that the successfully cloned three *CPK* genes (*AcoCPK1*, *AcoCPK3 and AcoCPK6*) belonged to Group I subfamily and we further focused on these three *CPK* genes. Subcellular locations of three CPK genes (*AcoCPK1*, *AcoCPK3 and AcoCPK6*) were identified, and we found their protein are mainly expressed at plasma membrane and small partial localized at nucleus (Fig.7), indicating that they are primarily soluble enzymes with the potential to target to the nucleus. Similar results had been found in *Arabidopsis CPK* family, with cytosolic and nuclear location for AtCPK3 and 4, a plasma membrane location for AtCPK7, 8, 9, 16, 21, and 28 and a peroxisome membrane location for AtCPK1 [53]. An increasing number of evidences have confirmed that *CPKs* play important roles in plant stress response and in related signaling pathways [6]. As shown in Figs. 5 and 6, some *CPK* genes shared similar expression patterns in response to a specific stress in the pineapple genome, for example, similar expression levels were observed among *AcoCPK1*, *AcoCPK3*, *AcoCPK11*, *AcoCPK14* and *AcoCPK17* in response to cold stress and *AcoCPK6* and *AcoCPK17* in response to mealybugs. Some genes can be induced by both biotic and abiotic stresses, such as *AcoCPK17* response to cold and mealybugs, *AcoCPK2* response to salt and mealybugs. Similar cross-talk between plant responses to biotic and abiotic stresses have been found to be mediated by *CPK* genes; for example, *PaCPK1* was activated by cold, wounding, and pathogen challenge [54], and *OsCPK12* functions in multiple signaling pathways, and it promotes tolerance to salt stress by reducing the accumulation of ROS and negatively modulates blast fungus resistance [18]. Thus, the cross-talk may help regulate the signaling network of *AcoCPK* in response to various types of stresses. Three genes, *AcoCPK1*, *AcoCPK3* and *AcoCPK6* were repressed by the abiotic stresses, and they also function in pathogen resistance (Figs.8 and 9). Based on the phylogenetic tree, *AcoCPK1* was clustered with *Arabidopsis AtCPK2,* and *AcoCPK3* and *AcoCPK6* was clustered with *Arabidopsis AtCPK1.* They have found that *AtCPK1* and *AtCPK2* were induced by drought, high-salt but not by low-temperature stress or heat stress [32, 55], while *AcoCPK3* and *AcoCPK6* were sensitive to drought and high-salt stress and they were also susceptible to *S. sclerotiorum*. When response to *S. sclerotiorum*, several marker genes related to ET, JA and ABA were suppressed indicating that phytohormone signaling pathway might be involved in the resistance response process. Genes with the same function are often closely related [49], indicating that *AcoCPK1*, *AcoCPK3* and *AcoCPK6* play important roles in different signal transduction pathways and in the adaption of pineapple to changeable environments and stresses. The distinct roles of pineapple and *Arabidopsis CPK* genes might be caused by the divergence of monocotyledones and dicotyledones during evolutionary.

## Conclusions

In summary, we identified 17 *CPK* genes in the pineapple genome that were distributed in eight chromosomes unevenly. According to the analysis of phylogenetic tree and gene structure, *AcoCPKs* are divided into four groups. Between pineapple and *Arabidopsis*, we identified 7 segmental duplication events and 5 syntenic blocks from CPKs, 8 between pineapple and rice. Expression profiles of *AcoCPKs* in response to various stresses implied their pivotal roles in participate in multiple signaling pathways. Our analyses provide an important foundation for understanding the potential roles of *AcoCPKs* in regulating pineapple response to biotic and abiotic stresses.

## Methods

### Identification of *CPKs* in pineapple and other species

Sequences from the pineapple genome database were downloaded from http://pineapple.angiosperms.org/pineapple/html/index.html. The 34 *Arabidopsis* CPKs protein sequences were obtained from http://www.Arabidopsis.org/. The rice and grape CPK sequences were downloaded from https://phytozome.jgi.doe.gov/pz/portal.html.

### Phylogenetic analysis of AcoCPK proteins

We performed sequence alignments of pineapple, rice, grape and *Arabidopsis* CPKs by using muscle (http://www.ebi.ac.uk/Tools/msa/muscle/). As previous study described, phylogenetic and molecular evolutionary analyses were generated using MEGA 6.0 software (http://www.megasoftware.net) and the RAxML program (http://www.phylo.org/) [49].

### Protein properties and sequence analyses

The ExPASy proteomics server (http://expasy.org/) [56] was used to identify the molecular weight and isoelectric points of predicted AcoCPK proteins. The MEME program (http://meme.nbcr. net/meme/cgi-bin/meme.cgi) was used to predict the conserved motifs in full-length pineapple CPK proteins with the following parameters: maximum number of motifs was 10 and the optimum width of motifs was set between 10 and 50 [57]. Gene structure analysis of *AcoCPK* was displayed with Gene Structure Display Server (http://gsds.cbi.pku.edu.cn/).

### Synteny analysis and chromosome localization

The syntenic blocks used for constructing a synteny analysis map within the pineapple genome and between the pineapple and *Arabidopsis* or rice genomes, were obtained by using BLASTP with the reference E < 1e-5 and top 3 matches [49]. All data used to analyze the expansion patterns of the *AcoCPK* family were shown in Additional file 6: Tables S3, Additional file 7: Table S4 and Table Additional file 8: S5. Diagrams were generated using the Circos program (version 0.69) (http://circos.ca/). Based on the comparative synteny map between the pineapple and Arabidopsis or rice genomes, the synonymous (Ks) and non-synonymous (Ka) nucleotide substitutions between orthologous gene pairs were calculated using ClustalW [58], PAL2NAL [59] and yn00 program of the bio-pipeline (https://github.com/ tanghaibao/ bio-pipeli ne).

### Expression profiles based on the estimation of expression levels from RNA-Seq data and qRT-PCR

We got the estimated expression levels, RPKM (reads per kilobase per million reads) values, for each *AcoCPKs* from 9 different tissues and developmental stages from https://de.iplantcollaborative.org/de/?type=data&folder=/iplant/home/cmwai/coge_data/Pineapple_tissue_RNAseq [30]. The expression level under abiotic stress treatments was obtained by quantitative real-time PCR (qRT-PCR) analysis.

### Plant materials and treatments

Pineapple (*Ananas comosus*) cultivar MD2 seedlings were grown in a greenhouse at 28 °C, 60–70 mmol photons m^− 2^ s^− 1^, a relative humidity of 70%, and a 16-h light/8-h dark photoperiod and the materials were provided by the pineapple breeding group in Fujian Agriculture and Forestry University.

Two-month old pineapple plants were treated with 400 mM NaCl and 400 mM mannitol to perform salt and drought stress, respectively, for 0, 2, 6, 12, 24 and 48 h. Heat treatment was carried out in a growth chamber at 45 °C, and cold treatment was performed in a growth chamber at 4 °C. For the biotic-stress treatment, two-month-old pineapple plants were infected with *mealybugs* (*Dysmicoccus brevipes*) [60]. The leaves were harvested at the indicated time points for the preparation of total RNA.

### RNA extraction and quantitative real-time PCR

We used Trizol method (Invitrogen, Carlsbad, CA, USA) to extract total RNA, and the PrimeScript RT-PCR kit (TaKaRa) was used to do the reverse-transcribed experiment. Real-time PCR was performed to analysis the relative transcript levels of selected genes according to the Bio-Rad Real-time PCR system (Foster City, CA, USA) and the SYBR Premix Ex Taq II system (TaKaRa Perfect Real Time), and the primers used has been listed in Additional file 15: Table S12. The qRT-PCR program was: 95 °C for 30 s; 40 cycles of 95 °C for 5 s and 60 °C for 34 s; 95 °C for 15 s [61, 62]. The relative transcript levels of the analyzed pineapple genes were normalized to the transcript levels of *AcoActin*.

### Vector constructs and pathogenicity assay

35S: AcoCPK1/3/6: GFP were generated by amplifying CDS sequence from wild-type pineapple cDNA using the primers listed in Additional file 14: Table S11. The PCR fragments were then cloned into the pENTR/D-TOPO vector (Invitrogen). pENTR clones were then recombined into the destination vector pGWB506 using LR Clonase II (Invitrogen). The three constructs were transformed in Arabidopsis Col-0 plants using the vacuum infiltration method [63]. Wild-type *S. sclerotiorum* isolate was cultured exclusively on minimal medium for 3 days before inoculation. An agar plug (about 2.5 mm in diameter), which contain the advancing edge of *S. sclerotiorum* mycelia, was used to inoculate *Arabidopsis* leaves. A single rosette leaf of 4- to 6-week-old *Arabidopsis* plants was inoculated for 12 h and then was harvested for measurement of lesion areas and RNA extraction.

### Subcellular localization

To examine the localization of *AcoCPK1/3/6* in planta, the plasmid containing *35S: AcoCPK1/3/6* construction transformed Agrobacterium tumefaciens GV3101 culture was resuspended in infiltration media before infiltration into 32d old leaves of *N. benthamiana*. 2 days later, leaf discs were observed of GFP under a confocal microscope (Leica TCS SP8X DLS) with laser 488 nm and 30% intensity, HYD %95 for GFP signal.

## Supplementary information

**Additional file 1: Figure S1.** 10 conserved motifs

**Additional file 2: Figure S2.** Expression profile of the pineapple *CPK* genes in different tissues and development stages

**Additional file 3 Figure S3** The relative expression level of CPK genes in transgenic Arabidopsis plants. **a** The expression level of *AcoCPK1* and the homologous *AtCPK11* in transgenic Arabidopsis plants of *OX-AcoCPK1*. **b** The expression level of *AcoCPK3* and the homologous *AtCPK1* in transgenic Arabidopsis plants of *OX-AcoCPK3*. **c** The expression level of *AcoCPK6* and the homologous *AtCPK2* in transgenic Arabidopsis plants of *OX-AcoCPK6*

**Additional file 4: Table S1.** Characteristics of CPKs in pineapple

**Additional file 5: TableS2.** Synteny blocks of *CPK* genes within pineapple genome

**Additional file 6: Table S3.** Synteny blocks of *CPK* genes between pineapple and Arabidopsis genomes

**Additional file 7: Table S4.** Synteny blocks of *CPK* genes between pineapple and rice genomes

**Additional file 8: Table S5.** Expression profiles of the pineapple *CPK* genes in different tissues

**Additional file 9: Table S6.** cis-elememts in *AcoCPKs* promoters

**Additional file 10: Table S7.** The expression profiles of the pineapple *CPK* genes after cold treatment

**Additional file 11: Table S8.** The expression profiles of the pineapple *CPK* genes after heat treatment

**Additional file 12: Table S9.** The expression profiles of the pineapple *CPK* genes after drought treatment

**Additional file 13: Table S10.** The expression profiles of the pineapple *CPK* genes after salt treatment

**Additional file 14: Table S11.** The expression profiles of the pineapple *CPK* genes after mealybugs infection

**Additional file 15: Table S12.** Primers used in this paper

## Data Availability

All data generated or analyzed during this study are included in this published article [and its supplementary information files].

## Abbreviations

Aco: Pineapple
At: *Arabidopsis thaliana*
CPK: Calcium-dependent protein kinase
GSDS: Gene Structure Display Server
HMM: Hidden Markov Models
MEGA: Molecular Evolutionary Genetics Analysis
MEME: Multiple Em for Motif Elicitation
NJ: Neighbor-joining
Os: *Oryza sativa*
qRT-PCR: Quantitative real-time PCR
RNA-seq: RNA sequencing
