# Supplementary figures and images for: Genome-wide investigation of calcium-dependent protein kinase gene family in pineapple: evolution and expression profiles during development and stress
Source: BMC Genomics. 2020 Jan 23;21:72. doi: 10.1186/s12864-020-6501-8 (PMC6979071; doi:10.1186/s12864-020-6501-8)

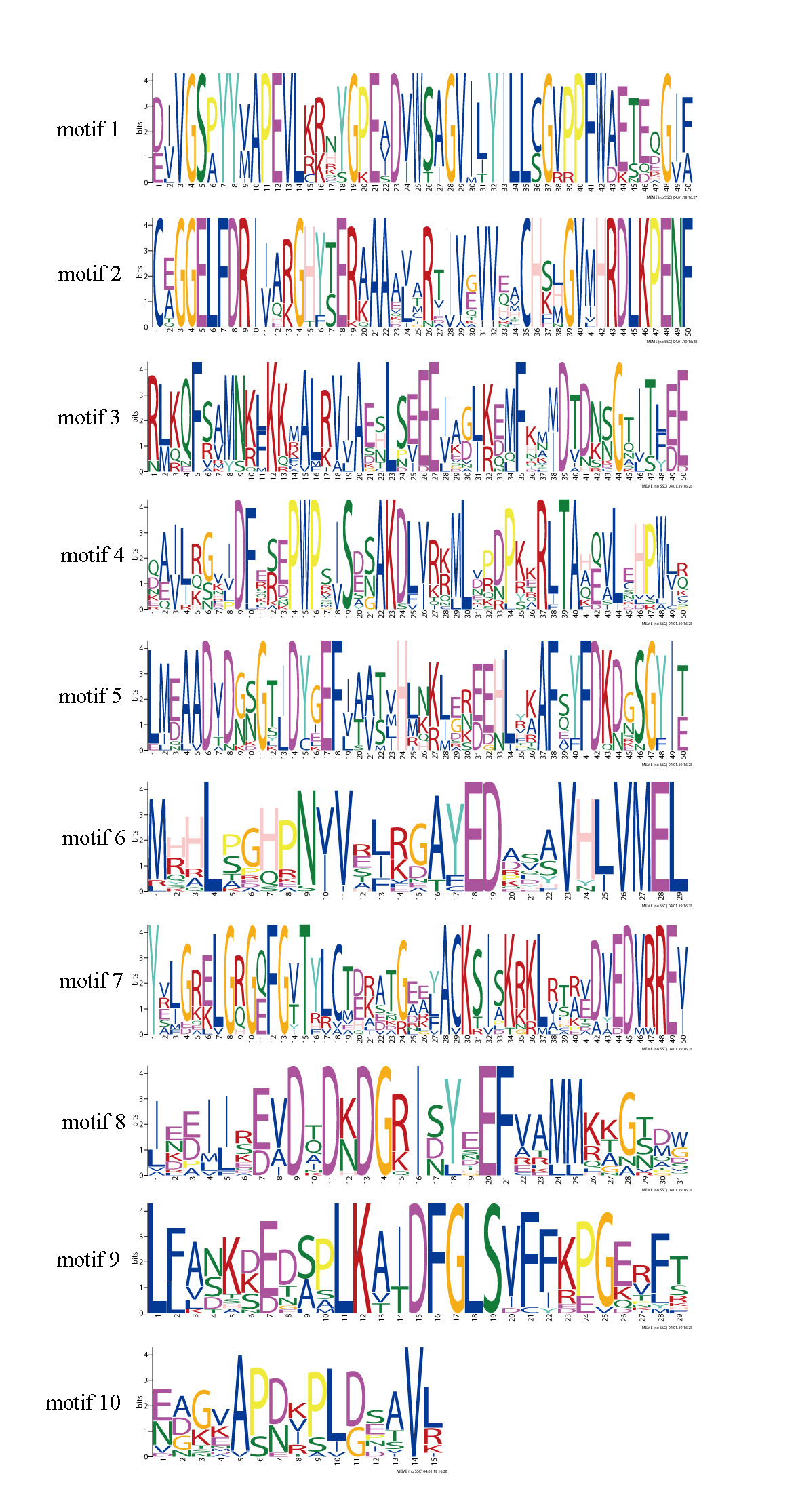

Supplement: Supplementary file 1 — Additional file 1: Figure S1. 10 conserved motifs [file 12864_2020_6501_MOESM1_ESM.jpg]

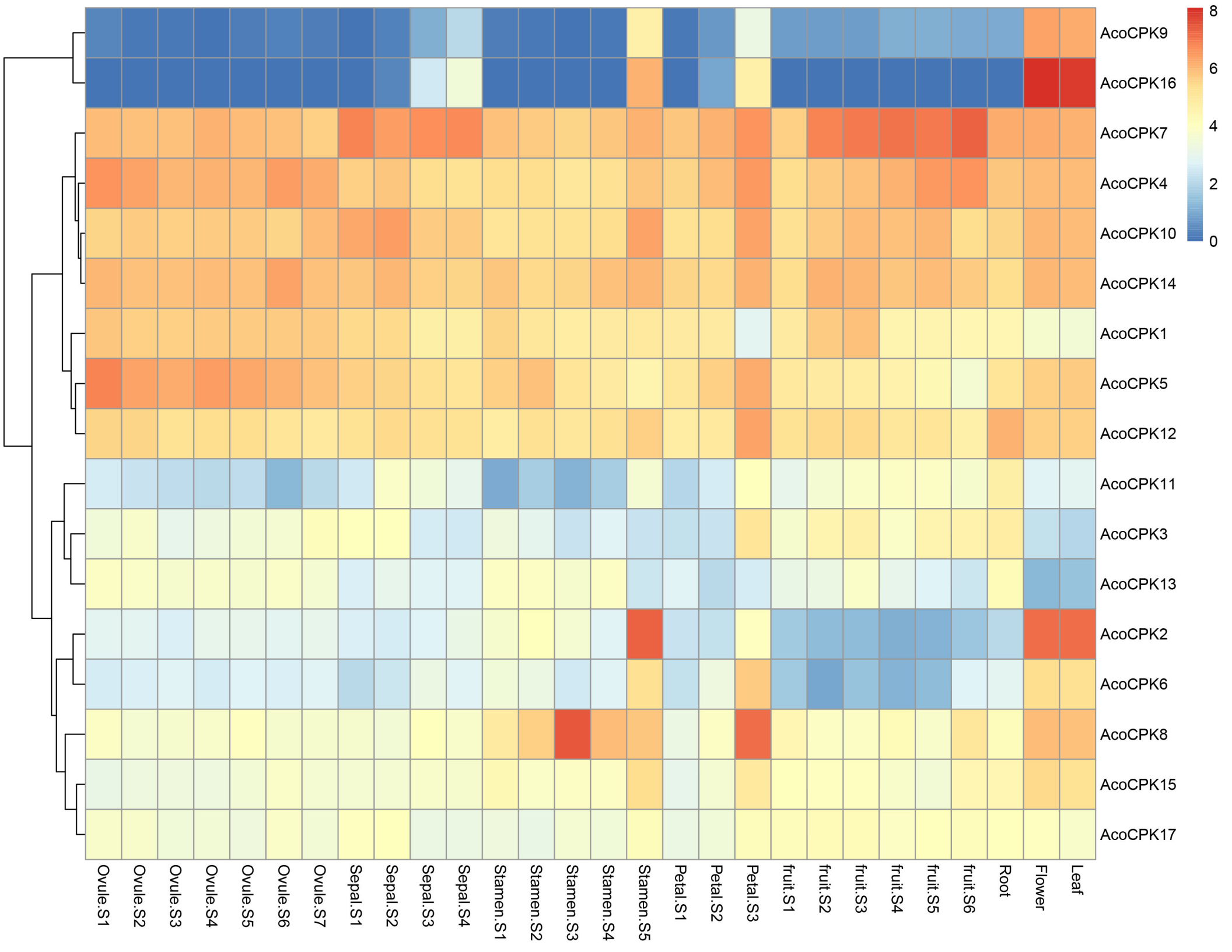

Supplement: Supplementary file 2 — Additional file 2: Figure S2. Expression profile of the pineapple CPK genes in different tissues and development stages [file 12864_2020_6501_MOESM2_ESM.pdf]

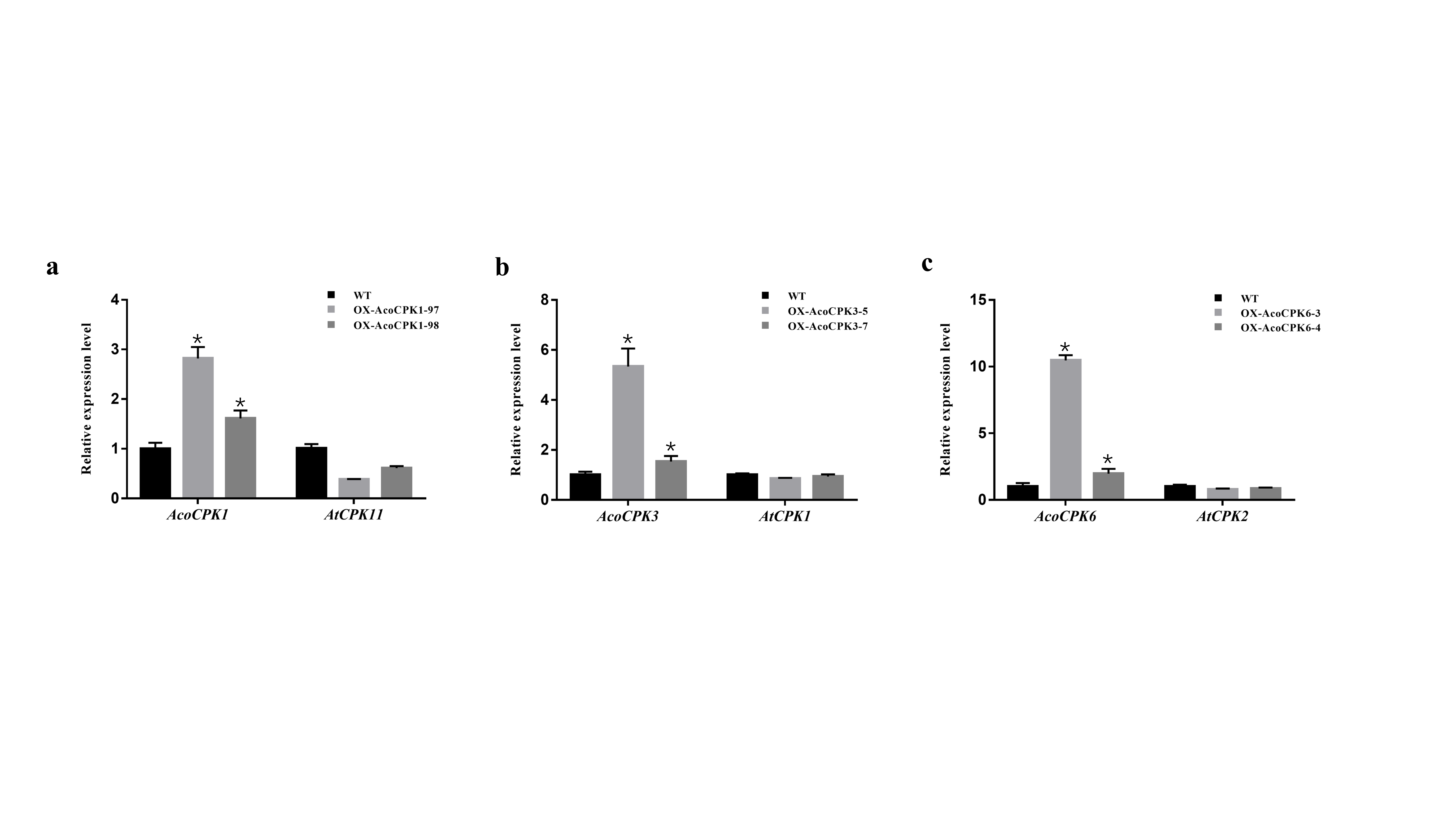

Supplement: Supplementary file 3 — Additional file 3 Figure S3 The relative expression level of CPK genes in transgenic Arabidopsis plants. a The expression level of AcoCPK1 and the homologous AtCPK11 in transgenic Arabidopsis plants of OX-AcoCPK1. b The expression level of AcoCPK3 and the homologous AtCPK1 in transgenic Arabidopsis plants of OX-AcoCPK3. c The expression level of AcoCPK6 and the homologous AtCPK2 in transgenic Arabidopsis plants of OX-AcoCPK6 [file 12864_2020_6501_MOESM3_ESM.tif]
